# Impact of the 21-Gene Oncotype DX Recurrence Score on Adjuvant Chemotherapy Decision-Making in Invasive Ductal Carcinoma: A Real-World Analysis From a Single Japanese Institution

**DOI:** 10.7759/cureus.113358

**Published:** 2026-07-25

**Authors:** Hirofumi Terakawa, Yuki Kurokawa, Ryosuke Mohri, Miki Hirata, Noriyuki Inaki

**Affiliations:** 1 Gastrointestinal Surgery/Breast Surgery, Kanazawa University Hospital, Kanazawa, JPN

**Keywords:** cancer chemotherapy, invasive ductal breast carcinoma, oncotype dx®, oncotype dx recurrence score, real-world evidence

## Abstract

Background

The 21-gene Oncotype DX Recurrence Score (RS) assay (Exact Sciences Corporation, Madison, WI) is a genomic test used to inform adjuvant chemotherapy decisions in hormone receptor (HR)-positive, human epidermal growth factor receptor 2 (HER2)-negative early breast cancer. In Japan, the assay has been reimbursed under national health insurance since September 2023, yet institution-level real-world data restricted to invasive ductal carcinoma (IDC) remain sparse. We therefore characterized the impact of the RS on adjuvant chemotherapy decisions in patients with IDC at our institution.

Methods

We conducted a single-center, retrospective, observational study of consecutive patients with HR-positive, HER2-negative IDC who underwent Oncotype DX testing at Kanazawa University Hospital between April 2022 and April 2026. Patients who received neoadjuvant systemic therapy were excluded a priori. Pathology was assessed locally rather than through external central review; estrogen receptor (ER), progesterone receptor (PgR), HER2, and Ki-67 were evaluated in accordance with the American Society of Clinical Oncology (ASCO)/College of American Pathologists (CAP) guidelines and the recommendations of the International Ki-67 in Breast Cancer Working Group. Clinical risk was dichotomized using the Adjuvant! Online version 8.0 simplified criteria (developed by Peter M. Ravdin, MD, PhD, at the University of Texas Health Science Center at San Antonio, TX) adopted in the MINDACT trial, and genomic risk was dichotomized at the TAILORx-defined threshold (RS ≤ 25 vs. RS ≥ 26). The primary endpoints were the clinico-genomic discordance rate and the actual rate of adjuvant chemotherapy administration or omission. Ninety-five percent Wilson score confidence intervals (CIs) were calculated for the principal proportions.

Results

Of 140 consecutive patients tested, 102 were eligible for analysis after exclusion of non-IDC histology (n = 24), missing histological grade (n = 12), an unevaluable RS result (n = 1), and incomplete clinical staging (n = 1). The median age was 55 years (range, 37-78); 59.8% of patients were postmenopausal, and 62.7% were node-negative. The median RS was 16 (range, 0-51); 17 patients (16.7%; 95% CI, 10.6-25.2) had an RS ≥ 26. Sixty-six patients (64.7%) were classified as clinical high risk and 36 (35.3%) as clinical low risk. The overall clinico-genomic discordance rate was 57.8% (59 of 102; 95% CI, 48.1-67.0) and was driven predominantly by the clinical high/RS low-intermediate combination (52.9%, 54 of 102; 95% CI, 43.3-62.4); the opposite, clinical low/RS high pattern accounted for only 4.9% (five of 102; 95% CI, 2.1-11.0). Adjuvant chemotherapy was withheld in 51 of 66 clinical high-risk patients (77.3%; 95% CI, 65.7-85.7) and in 50 of 54 clinical high/RS low-intermediate patients (92.6%; 95% CI, 82.4-97.1). Overall, 17 of 102 patients (16.7%; 95% CI, 10.6-25.2) received adjuvant chemotherapy.

Conclusions

In this Japanese single-institution IDC cohort, approximately three in five patients showed discordance between clinical and genomic risk, with reclassification overwhelmingly directed toward de-escalation. Oncotype DX testing was associated with the omission of adjuvant chemotherapy in more than three-quarters of clinically high-risk IDC patients, supporting its substantial influence on adjuvant treatment decision-making in routine Japanese clinical practice.

## Introduction

Hormone receptor (HR)-positive, human epidermal growth factor receptor 2 (HER2)-negative early breast cancer accounts for approximately 70% of all newly diagnosed breast cancers and is the most prevalent molecular subtype [1,2]. Adjuvant endocrine therapy remains the cornerstone of systemic treatment in this subtype, whereas the incremental benefit of adjuvant chemotherapy is modest and confined to a clinically and biologically defined subset [2,3]. Reliable identification of the patients who truly benefit from chemotherapy is therefore essential to balance the avoidance of overtreatment with optimal oncologic outcomes [4].

The 21-gene Oncotype DX Recurrence Score (RS) assay is a prognostic and predictive tool whose clinical utility for guiding adjuvant chemotherapy decisions has been prospectively validated in node-negative (N0) and one- to three-node-positive (N1) disease through the Trial Assigning Individualized Options for Treatment (TAILORx) [1,5] and the Rx for Positive Node, Endocrine Responsive Breast Cancer (RxPONDER) trial [6]. The Microarray In Node-negative and 1 to 3 positive lymph node Disease may Avoid ChemoTherapy (MINDACT) trial further showed that adjuvant chemotherapy can be safely omitted in patients judged clinically high risk but genomically low risk, establishing the integrated clinico-genomic risk paradigm as a contemporary standard for adjuvant decision-making [7,8]. These principles are reflected in the current American Society of Clinical Oncology (ASCO) biomarker guideline [3] and in the St. Gallen International Consensus [9].

In Japan, the Oncotype DX assay has been reimbursed by the national health insurance system since September 2023, and its use in routine clinical practice has expanded rapidly. Nevertheless, real-world Japanese data - particularly studies restricted to invasive ductal carcinoma (IDC) that quantify both the rate of clinico-genomic discordance and the impact on chemotherapy choice - remain limited.

In the present study, “clinico-genomic discordance” is defined as a mismatch between the dichotomized clinical risk (Adjuvant! Online version 8.0 criteria (developed by Peter M. Ravdin, MD, PhD, at the University of Texas Health Science Center at San Antonio, TX), as adopted in MINDACT) and the dichotomized genomic risk based on the Oncotype DX RS (TAILORx threshold: RS ≤ 25 vs. RS ≥ 26). We therefore analyzed consecutive patients with IDC who underwent Oncotype DX testing at our institution, with two primary objectives: (i) to quantify the discordance between clinical risk - defined according to the MINDACT/Adjuvant! Online version 8.0 criteria - and RS-based genomic risk; and (ii) to characterize the impact of the RS on final adjuvant chemotherapy decisions in routine Japanese practice. We hypothesized that, in a homogeneous Japanese IDC cohort following national reimbursement, a substantial proportion of clinically high-risk patients would be reclassified as genomic low/intermediate risk, and that Oncotype DX testing would be associated with a meaningful reduction in the use of adjuvant chemotherapy compared with a clinical risk-only approach.

## Materials and methods

Study design and patients

We undertook a single-center, retrospective, observational study at the Department of Breast Surgery, Kanazawa University Hospital. Consecutive patients with estrogen receptor (ER)-positive, HER2-negative early breast cancer who underwent Oncotype DX testing (Exact Sciences Corporation, Madison, WI) between April 2022 and April 2026 were reviewed. Eligibility required pure IDC histology and a Nottingham histological grade [10] documented in the pathology report. Patients with invasive lobular carcinoma, mucinous carcinoma, invasive papillary carcinoma, or mixed histology (e.g., IDC combined with invasive lobular carcinoma, or IDC with a mucinous component) were excluded because of their biological heterogeneity. Patients who received any form of neoadjuvant systemic therapy - either neoadjuvant chemotherapy or neoadjuvant endocrine therapy - were excluded a priori, since the RS was evaluated in this study as a tool for guiding adjuvant therapy decisions in chemotherapy-naïve disease.

The study was approved by the Institutional Review Board of Kanazawa University Hospital (approval number 115084). Given the retrospective design, the requirement for written informed consent was waived, and an opt-out approach was adopted in accordance with institutional policy.

Institutional criteria for ordering Oncotype DX testing

To allow readers to evaluate selection bias arising from test indication, we describe explicitly the institutional criteria that were applied during the study period. Oncotype DX testing was offered to and discussed with patients who met all of the following conditions: ER-positive, HER2-negative early breast cancer with zero macroscopic axillary lymph node metastases, micrometastasis only, or one to three macrometastases. For premenopausal women with node-positive disease, adjuvant chemotherapy was generally recommended, but Oncotype DX testing was additionally offered when the patient requested it. No strict tumor-size threshold was applied; in practice, invasive tumors with an infiltrative component of at least 2 mm were predominantly tested. Special histological subtypes were not excluded a priori from testing at the institutional level, although such cases were excluded from the present analysis, as described above.

Pathology evaluation

All pathology specimens were reviewed at our institution by board-certified breast pathologists; no central pathology review by an external laboratory was performed. ER and PgR status were determined by immunohistochemistry (IHC) in accordance with the ASCO/CAP guideline [11], with tumors considered positive when at least 1% of tumor cell nuclei showed staining. Both the percentage of positively stained cells and the J-score were reported. HER2 status was assessed according to the 2018 ASCO/CAP focused update [12] using the Ventana anti-HER2/neu (4B5) rabbit monoclonal primary antibody (Roche Diagnostics, Tucson, AZ); equivocal (IHC 2+) cases were reflexed to dual in situ hybridization (DISH) for definitive classification. The Ki-67 labeling index was determined by counting at least 500 invasive tumor cells in hot-spot regions, consistent with the recommendations of the International Ki-67 in Breast Cancer Working Group [13]. In two cases, the Ki-67 index was reported semi-quantitatively as “< 1%” without a precise numerical value; these two cases were treated as missing for the Ki-67 summary statistics but were retained for all other endpoints, as Ki-67 was not used in the clinical or genomic risk classification.

Axillary nodal assessment

Patients in whom axillary lymph node metastasis was confirmed preoperatively by imaging (ultrasonography or computed tomography) together with fine-needle aspiration cytology underwent upfront axillary lymph node dissection. All remaining patients underwent sentinel lymph node biopsy. In mastectomy cases, sentinel nodes were examined intraoperatively by frozen section, and completion axillary dissection was added when macrometastatic deposits of at least 2 mm were identified. In breast-conserving surgery, sentinel nodes were not submitted for intraoperative analysis; final histopathology served as the diagnostic standard, and axillary dissection was omitted for up to two macrometastatic sentinel nodes in accordance with contemporary practice informed by the ACOSOG Z0011 trial [14]. Whenever feasible, a minimum of three sentinel nodes were sampled. Sentinel lymph nodes were serially sectioned at 2-mm intervals for histopathological evaluation.

Data collection

The following variables were extracted from the electronic medical records: age, menopausal status, pathological tumor size (pT, in millimeters), number of axillary lymph node metastases (pN), Nottingham histological grade (G1, G2, or G3), immunohistochemical expression of ER and PgR, HER2 status, Ki-67 labeling index, Oncotype DX RS, and receipt of adjuvant chemotherapy. In every included patient, the RS was available before the final adjuvant treatment decision and was explicitly incorporated into the subsequent shared decision-making discussion. Of note, patients with strong pre-existing preferences for or against chemotherapy were generally not offered the assay in the first place; this practice may have selected for a population in whom the RS could meaningfully influence the decision.

Clinical risk classification

Clinical risk was dichotomized using the Adjuvant! Online version 8.0 simplified criteria adopted in the MINDACT trial [7]. Patients meeting any one of the following criteria were classified as clinical low risk: (i) N0, pT ≤ 20 mm, grade 1; (ii) N0, pT ≤ 10 mm, grade 2; (iii) N0, pT ≤ 10 mm, grade 3; or (iv) N1 (one to three positive nodes), pT ≤ 20 mm, grade 1. All remaining patients, including any with N2 disease, were classified as clinical high risk. Individual Adjuvant! Online numerical risk scores were not calculated. This framework was selected for two reasons. First, it is the most widely used dichotomized clinical risk stratification in contemporary integrated clinico-genomic analyses, including the MINDACT trial [7,8] and the clinical-genomic secondary analysis of TAILORx [5]; its use therefore enables direct comparison of our findings with those of the major reference trials. Second, it explicitly accommodates the one- to three-node-positive (N1) subgroup, which constituted 37.3% of our cohort. The binary clinical-risk classification displayed on the official Oncotype DX Breast RS report (Exact Sciences Corporation; defined as low clinical risk when the sum of histological grade and tumor size in centimeters is ≤ 4, and high clinical risk when the sum is > 4) corresponds essentially to the simplified criteria used in the clinical-genomic secondary analysis of TAILORx [5] and applies primarily to node-negative disease; consequently, we adopted the MINDACT framework as the more appropriate option for a mixed node-negative and node-positive cohort. The limitations of applying any single clinical-risk framework to an Oncotype DX cohort are addressed in the Discussion.

Genomic risk classification

Oncotype DX RS values were dichotomized according to the TAILORx framework [1]: RS ≤ 25 was classified as low/intermediate genomic risk, and RS ≥ 26 as high genomic risk.

Adjuvant treatment protocols and decision-making process

Final adjuvant treatment decisions were made through shared decision-making between the treating breast surgeon and the patient, integrating the Oncotype DX RS, clinicopathological risk factors, age and menopausal status, comorbidities, and patient preference. During the study period, our institution had not yet adopted an RS-based regimen-selection algorithm-for example, the framework recently proposed by Chen et al., in which patients with RS ≥ 31 derived greater benefit from an anthracycline-and-taxane sequential regimen (T-AC) than from docetaxel plus cyclophosphamide (TC) in a large real-world cohort of genomic high-risk, node-negative, HR-positive/HER2-negative breast cancer [15]. When cytotoxic chemotherapy was recommended, an anthracycline-and-taxane sequential regimen was preferred over TC. In patients aged ≤ 65 years without significant comorbidities, a dose-dense schedule consisting of four cycles of epirubicin (E; 90 mg/m²) plus cyclophosphamide (C; 600 mg/m²) administered every two weeks with pegfilgrastim support, followed by four cycles of dose-dense paclitaxel (175 mg/m² every two weeks), was used as the standard regimen (ddEC → ddPTX), on the basis of accumulating evidence that dose-dense adjuvant regimens improve breast cancer-specific recurrence-free survival across major subgroups, including luminal disease [16,17]. For patients aged > 65 years or with clinically relevant comorbidities in whom dose-dense scheduling was considered inappropriate, four cycles of conventional every-three-week EC (epirubicin 90 mg/m² plus cyclophosphamide 600 mg/m²) followed by four cycles of triweekly docetaxel (75 mg/m² every three weeks) were administered instead. TC was reserved for patients with contraindications to, or intolerance of, anthracyclines. Prospective incorporation of RS-guided regimen selection - including consideration of T-AC for RS ≥ 31 and TC for lower RS categories in whom cytotoxic therapy is indicated - is planned in future institutional protocols.

Adjuvant endocrine therapy was prescribed to all patients with HR-positive disease. For premenopausal patients, tamoxifen combined with a luteinizing hormone-releasing hormone agonist (LHRHa) was administered; ovarian function suppression combined with an aromatase inhibitor (AI + LHRHa) was not used, as this combination is not reimbursed under the Japanese national health insurance system for adjuvant use in premenopausal early breast cancer. For postmenopausal patients, an aromatase inhibitor was used. Duration and any subsequent switch or extended endocrine therapy were individualized on the basis of tolerability and recurrence risk.

Beyond the RS, factors that influenced the final chemotherapy decision included age (with particular attention to premenopausal women aged ≤ 50 years with an RS of 16-25, in whom an absolute chemotherapy benefit has been suggested by the TAILORx clinical-genomic secondary analysis [5]), nodal status, Ki-67 labeling index, histological grade, comorbidities, and patient preference after informed discussion.

Endpoints and definition of adjuvant chemotherapy

The primary endpoints were (i) the clinico-genomic discordance rate between clinical and genomic risk classifications and (ii) the rates of administration and omission of adjuvant chemotherapy (decision impact). The secondary endpoint was the chemotherapy administration rate within each of the four clinical risk × genomic risk strata. Chemotherapy data reflected the treatment that was actually received, rather than the initial physician recommendation. Patients were considered to have received chemotherapy if they were treated with any cytotoxic regimen containing an anthracycline, a taxane, or cyclophosphamide/methotrexate/5-fluorouracil (CMF). Single-agent oral fluoropyrimidine (S-1) was not regarded as cytotoxic chemotherapy in this analysis: in the POTENT trial [18], S-1 was investigated as an addition to endocrine therapy in patients in whom intravenous cytotoxic chemotherapy was not indicated, and S-1 is therefore not used in routine practice as a substitute for intravenous chemotherapy. Furthermore, the timing of Japanese national insurance reimbursement of adjuvant S-1 differed from that of Oncotype DX during the study period. Accordingly, patients who received only adjuvant S-1 in addition to endocrine therapy and patients who declined the recommended cytotoxic chemotherapy were both classified as “chemotherapy not administered”; the numerical breakdown of these categories is reported in the Results.

Statistical analysis

Continuous variables are presented as the median (range), and categorical variables as counts and percentages. Ninety-five percent Wilson score CIs were calculated for the principal proportions, including clinico-genomic discordance, chemotherapy administration, and chemotherapy omission, both overall and within strata. The Wilson method was chosen because it remains stable for proportions close to 0 or 1 and for small subgroup sample sizes. No formal between-group hypothesis testing was performed; the analysis was descriptive. Missing data were handled exclusively by exclusion at the patient-selection stage, as detailed in the patient flow diagram (presented in the first figure in the Results section); no imputation was performed. After these exclusions, the analytic dataset for the primary endpoints contained no missing values. Ki-67 was missing in two patients and is reported as such in the first table in the Results section, without affecting calculation of the primary or secondary endpoints. All analyses and figure preparation were performed in R (version 4.5.0, R Foundation for Statistical Computing, Vienna, Austria) [19], using the dplyr, gtsummary, ggplot2, and ggalluvial packages.

## Results

Patient flow and characteristics

**Figure 1:**
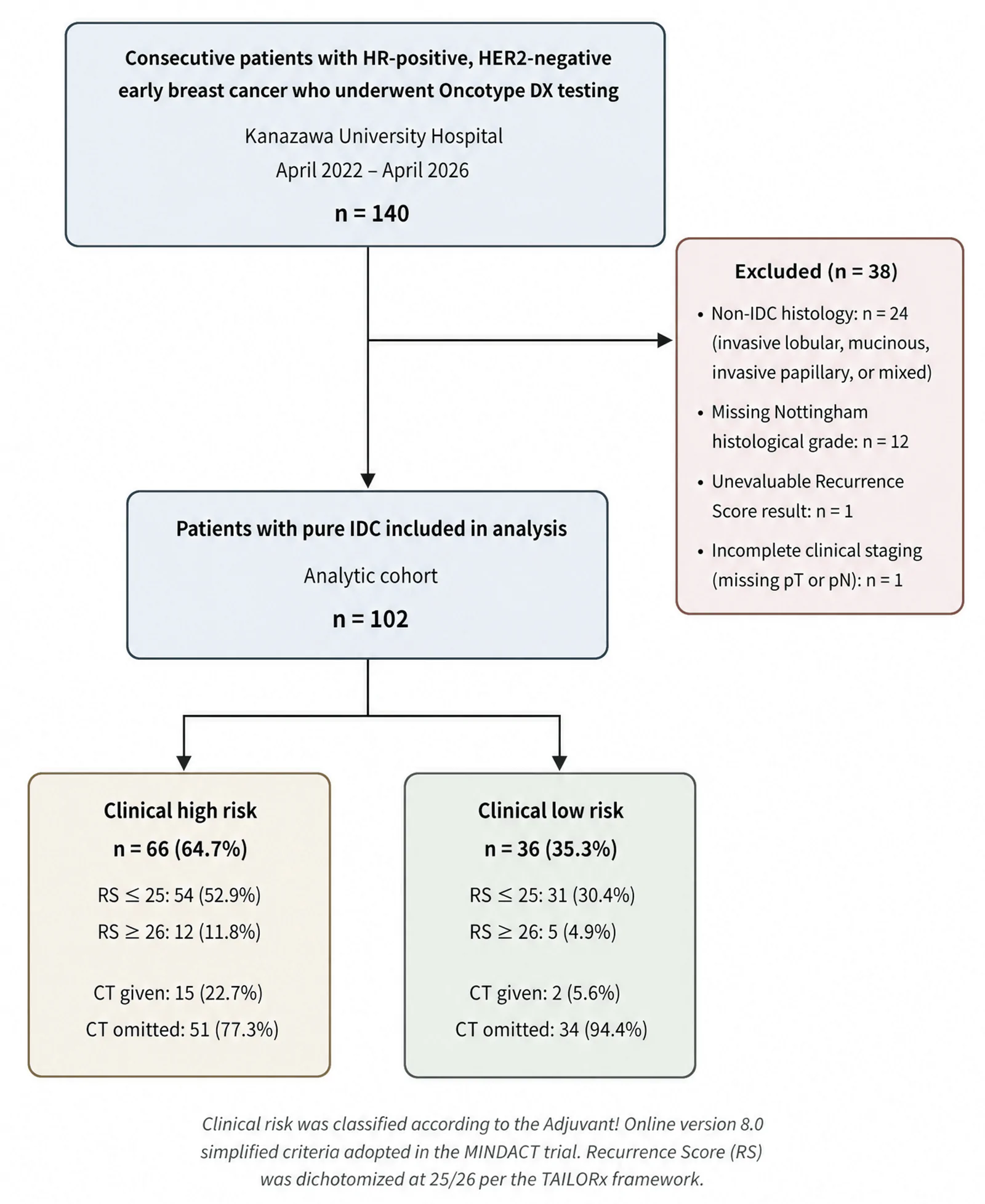
Patient selection flow diagram Of 140 consecutive patients with HR-positive, HER2-negative early breast cancer who underwent Oncotype DX testing at Kanazawa University Hospital between April 2022 and April 2026, 38 were excluded: 24 with non-IDC histology (invasive lobular, mucinous, invasive papillary, or mixed subtypes), 12 without a documented Nottingham histological grade (tested predominantly in the early study period, before institutional reporting was standardized to include the histological grade in addition to nuclear grade), one with an unevaluable RS result, and one with incomplete pT or pN data precluding clinical risk classification. Patients receiving neoadjuvant systemic therapy were excluded a priori at the inclusion stage. The remaining 102 patients with pure IDC were analyzed. Excluded patients did not differ systematically from the analytic cohort in age, tumor size, or nodal status. CT, cytotoxic chemotherapy; HR, hormone receptor; HER2, human epidermal growth factor receptor 2; IDC, invasive ductal carcinoma; RS, Recurrence Score This figure is generated using Python (version 3.11, Python Software Foundation, Wilmington, DE) with the Matplotlib and Plotly libraries.

Of 140 consecutive patients who underwent Oncotype DX testing during the study period, 38 were excluded: 24 with non-IDC histology (invasive lobular, mucinous, invasive papillary, or mixed subtypes), 12 without a documented histological grade, one with an unevaluable RS result, and one with missing pT or pN data that precluded clinical risk classification-leaving 102 patients eligible for analysis (Figure 1). The 12 patients excluded for a missing Nottingham grade were tested in the early portion of the study period (predominantly in 2022), when our pathology reports recorded the nuclear grade only; following an internal request to the Department of Pathology in mid-2022 to additionally record the Nottingham histological grade, no further such exclusions occurred. These excluded patients did not differ systematically from the analytic cohort in age, tumor size, nodal status, or Ki-67. Accordingly, the exclusion is best interpreted as a temporal documentation artifact rather than a clinically meaningful selection bias; nevertheless, residual selection effects cannot be fully excluded and are addressed among the limitations.

**Figure 2:**
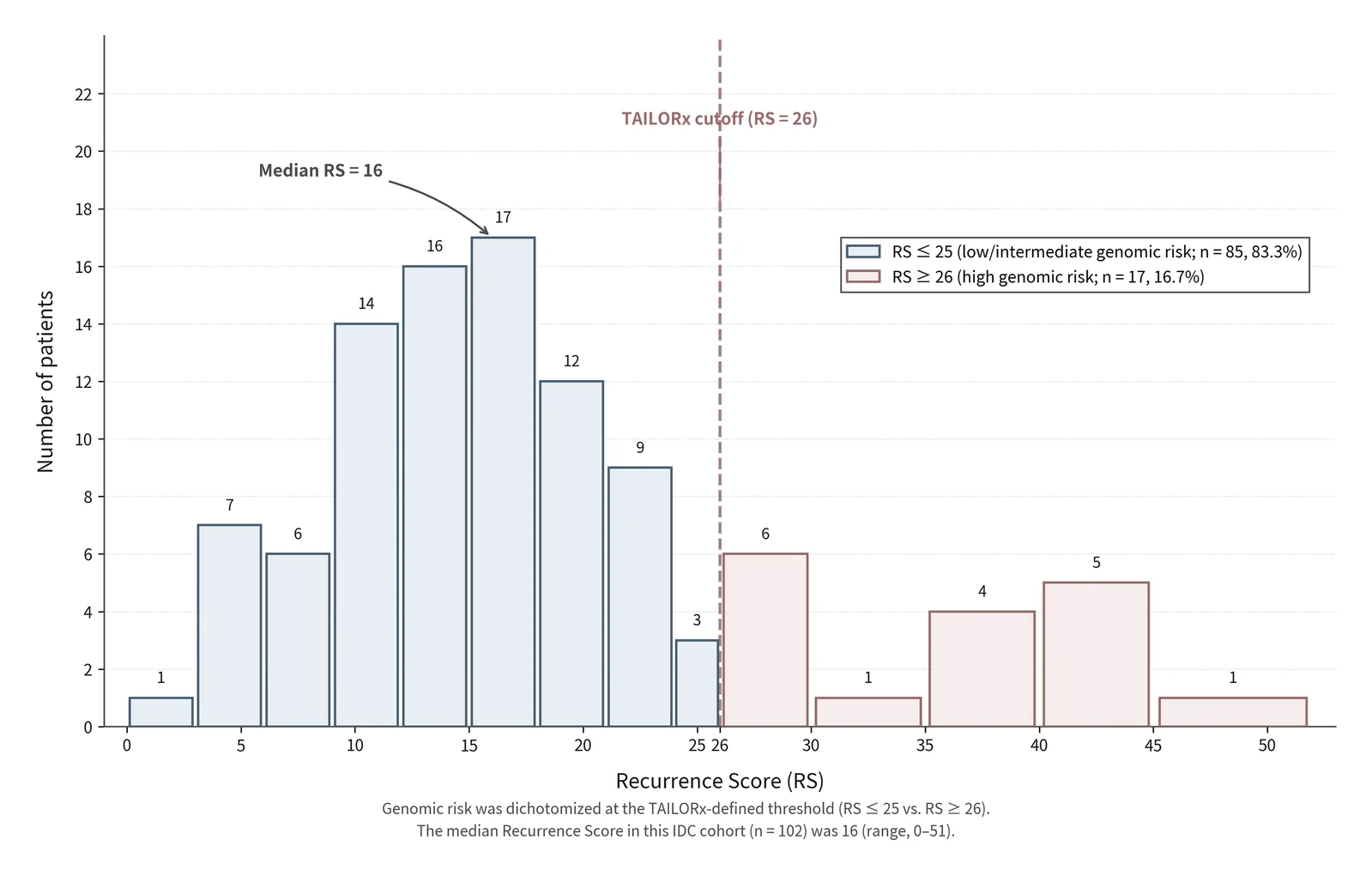
Distribution of the Recurrence Score (RS) in the invasive ductal carcinoma (IDC) cohort (n = 102) Bars represent the number of patients in each RS bin; the red dashed line marks the Trial Assigning Individualized Options for Treatment (TAILORx) cutoff (RS = 26). The median RS was 16 (range, 0-51), and 17 patients (16.7%) were classified as RS high (RS ≥ 26). This figure is generated using Python (version 3.11, Python Software Foundation, Wilmington, DE) with the Matplotlib and Plotly libraries.

The clinical and pathological characteristics of the 102 eligible patients are summarized in Table 1, and the distribution of the RS is shown in Figure 2. The median age was 55 years (range, 37-78); 61 of 102 patients (59.8%) were postmenopausal, and 64 of 102 (62.7%) had node-negative disease. The median tumor size was 16 mm, grade 2 tumors comprised 53 of 102 cases (52.0%), and the median Ki-67 labeling index was 12.7% (range, 1.0-95.0; n = 100, with two semi-quantitative “< 1%” results treated as missing). The median RS was 16 (range, 0-51); 17 patients (16.7%; 95% CI, 10.6-25.2) had an RS ≥ 26 and were therefore classified as RS high.

**Table 1:** Patient and tumor characteristics (n = 102) Confidence intervals are 95% Wilson score intervals. °In two patients the Ki-67 index was reported as “< 1%” without a precise numerical value; these two cases were excluded from the Ki-67 summary statistics (Ki-67 n = 100) but retained for all other endpoints. ^†^Patients who received only adjuvant S-1 in addition to endocrine therapy (n = 2) and one patient who declined the recommended cytotoxic regimen were classified as “cytotoxic chemotherapy not administered,” consistent with the POTENT trial framework, in which S-1 was investigated as an adjunct to endocrine therapy rather than as a substitute for intravenous cytotoxic chemotherapy. RS, Recurrence Score; CI, confidence interval; S-1, single-agent oral fluoropyrimidine

Characteristic	Value (n = 102)
Age (years), median (range)	55 (37-78)
Menopausal status, n (%)	
Premenopausal	32 (31.4)
Perimenopausal	9 (8.8)
Postmenopausal	61 (59.8)
Tumor size (mm), median (range)	16 (5-98)
Nodal status, n (%)	
N0	64 (62.7)
N1+ (pN1, 1-3 metastatic nodes)	38 (37.3)
Histological grade, n (%)	
G1	35 (34.3)
G2	53 (52.0)
G3	14 (13.7)
Ki-67 (%), median (range)°	12.7 (1.0-95.0)
Recurrence Score, median (range)	16 (0-51)
RS high (≥ 26), n (%) (95% CI)	17 (16.7) (10.6-25.2)
Clinical risk, n (%) (95% CI)	
Clinical low	36 (35.3) (26.7-45.0)
Clinical high	66 (64.7) (55.0-73.3)
Adjuvant systemic therapy, n (%) (95% CI)†	
Cytotoxic chemotherapy administered	17 (16.7) (10.6-25.2)
Cytotoxic chemotherapy not administered	85 (83.3) (74.8-89.4)
Endocrine therapy alone	82 (80.4)
Endocrine therapy plus S-1	2 (2.0)
Patient-declined cytotoxic regimen (endocrine therapy alone)	1 (1.0)

Clinico-genomic discordance

**Figure 3:**
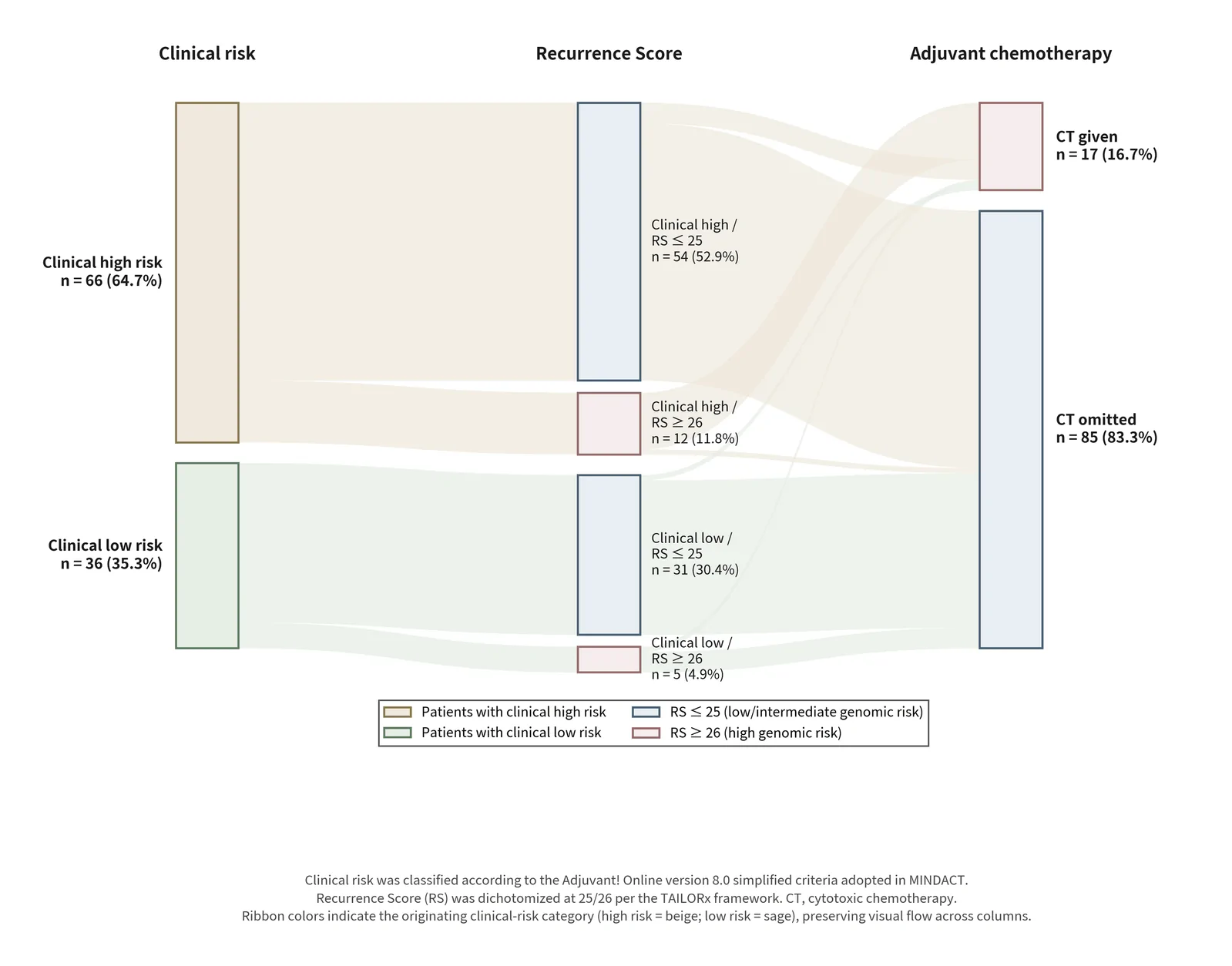
Sankey diagram Sankey diagram illustrating patient flow from clinical risk (Adjuvant! Online version 8.0 criteria) to RS classification and to the final adjuvant chemotherapy decision in the IDC cohort (n = 102). The majority of clinically high-risk patients were reclassified as RS low/intermediate and avoided chemotherapy, whereas patients classified as RS high predominantly received chemotherapy. The Sankey diagram was generated with the ggalluvial package (version 0.12.5) in R (version 4.5.0, R Foundation for Statistical Computing, Vienna, Austria). CT, cytotoxic chemotherapy; IDC, invasive ductal carcinoma; MINDACT, Microarray In Node-negative and 1 to 3 positive lymph node Disease may Avoid ChemoTherapy; RS, Recurrence Score; TAILORx, Trial Assigning Individualized Options for Treatment This figure is generated using Python (version 3.11, Python Software Foundation, Wilmington, DE) with the Matplotlib and Plotly libraries.

Application of the Adjuvant! Online version 8.0 criteria classified 66 patients (64.7%; 95% CI, 55.0-73.3) as clinical high risk and 36 (35.3%; 95% CI, 26.7-45.0) as clinical low risk. The overall clinico-genomic discordance rate was 57.8% (59 of 102; 95% CI, 48.1-67.0). The clinical high/RS low-intermediate group accounted for the majority of discordant cases (52.9%, 54 of 102; 95% CI, 43.3-62.4), whereas the clinical low/RS high group represented only 4.9% (5 of 102; 95% CI, 2.1-11.0). Discordance in this cohort was therefore driven predominantly by downward genomic reclassification of clinically high-risk patients (Figure 3). The full cross-tabulation of clinical risk, RS subgroup, and final chemotherapy decision is presented in Table 2.

**Table 2:** Cross-tabulation of clinical risk, RS, and final adjuvant chemotherapy decision (n = 102), with 95% Wilson confidence intervals for chemotherapy omission rates Clinical risk was defined according to the Adjuvant! Online version 8.0 criteria adopted in the MINDACT trial. RS was dichotomized at 25/26 according to the TAILORx framework. Confidence intervals are 95% Wilson score intervals for the proportion of patients in whom chemotherapy was omitted. CI: confidence interval; CT: cytotoxic chemotherapy; RS: Recurrence Score

Clinical risk	RS subgroup	n	% of total	CT given, n (%)	CT omitted, n (%) (95% CI)
Clinical high	Overall	66	64.7	15 (22.7)	51 (77.3) (65.7-85.7)
	RS low/intermediate (≤ 25)	54	52.9	4 (7.4)	50 (92.6) (82.4-97.1)
	RS high (≥ 26)	12	11.8	11 (91.7)	1 (8.3) (1.5-35.4)
Clinical low	Overall	36	35.3	2 (5.6)	34 (94.4) (81.9-98.5)
	RS low/intermediate (≤ 25)	31	30.4	1 (3.2)	30 (96.8) (83.8-99.4)
	RS high (≥ 26)	5	4.9	1 (20.0)	4 (80.0) (37.6-96.4)
Total		102	100.0	17 (16.7)	85 (83.3) (74.8-89.4)

Impact on chemotherapy decisions

Among the 66 clinical high-risk patients, only 15 (22.7%) ultimately received adjuvant chemotherapy, whereas 51 (77.3%; 95% CI, 65.7-85.7) did not. In the clinical high/RS low-intermediate subgroup (n = 54), chemotherapy was omitted in 50 patients (92.6%; 95% CI, 82.4-97.1), constituting the largest de-escalation subset in the cohort. Conversely, in the clinical high/RS high subgroup (n = 12), 11 patients (91.7%) received chemotherapy. Among the 36 clinical low-risk patients, chemotherapy was administered to only two (5.6%) (Table 2). Among the 38 patients with node-positive (pN1-3) disease, five received and 33 were spared adjuvant chemotherapy; chemotherapy decisions in this subgroup were closely concordant with the RS, particularly in postmenopausal patients, consistent with the RxPONDER results [6]. In aggregate, only 17 of 102 IDC patients (16.7%; 95% CI, 10.6-25.2) received adjuvant chemotherapy, indicating that cytotoxic chemotherapy was avoided in 85 of 102 patients (83.3%). Within the 85 patients classified as “chemotherapy not administered,” 82 received endocrine therapy alone, two additionally received adjuvant S-1 in combination with endocrine therapy (in line with the POTENT framework), and one declined the recommended cytotoxic regimen and elected endocrine therapy alone.

## Discussion

Our analysis of real-world Oncotype DX testing at a Japanese single institution, restricted to patients with pure IDC and a documented Nottingham grade, yielded two principal findings. First, the discordance rate between Adjuvant! Online version 8.0-based clinical risk and Oncotype DX-based genomic risk reached 57.8% (95% CI, 48.1-67.0), with the great majority of discordant cases (52.9%) representing downward reclassification from clinical high to genomic low/intermediate risk. Second, even among clinically high-risk patients, adjuvant chemotherapy was ultimately omitted in 77.3% (95% CI, 65.7-85.7), with the clinical high/RS low-intermediate subgroup-in whom 92.6% of patients did not receive chemotherapy - constituting the principal driver of the decision impact of the assay in this cohort.

The MINDACT trial reported a five-year distant metastasis-free survival rate of 94.7% among patients with clinical high/genomic low risk in whom adjuvant chemotherapy was omitted, providing prospective randomized evidence supporting de-escalation of cytotoxic therapy in clinically high-risk early breast cancer [7,8]. However, the present study evaluated treatment-decision behavior rather than oncologic outcomes; we therefore did not assess whether the observed de-escalation is safe in terms of invasive disease-free or overall survival. Although the clinicopathological profile of patients in whom chemotherapy was omitted closely mirrored that of the MINDACT de-escalation cohort, any inference regarding the long-term safety of chemotherapy omission in the present cohort remains provisional and requires confirmation through extended follow-up and multicenter validation. These observations are consistent with the current ASCO biomarker guideline [3], the St. Gallen International Consensus [9], and the Japanese Breast Cancer Society clinical practice guidelines [20], all of which endorse the integration of Oncotype DX results with clinical risk in adjuvant decision-making.

The high clinical high-RS low-intermediate discordance rate observed in our cohort warrants cautious interpretation. The Adjuvant! Online version 8.0 simplified criteria adopted in MINDACT classify a large proportion of grade 2 tumors larger than 1 cm and virtually all node-positive tumors as clinical high risk, irrespective of Ki-67 and luminal-like biological features that more contemporary risk frameworks emphasize. Of note, the binary clinical-risk classification displayed on the official Oncotype DX Breast RS report (Exact Sciences Corporation) uses a slightly less restrictive threshold based on the sum of grade and tumor size (low risk if ≤ 4; high risk if > 4), corresponding to the simplified criteria of the TAILORx clinical-genomic secondary analysis [5]; this threshold is principally intended for node-negative disease. Consequently, the apparent magnitude of discordance is partly a function of the chosen risk-classification framework rather than an intrinsic biological inconsistency. Alternative frameworks-such as the St. Gallen risk groups [9], the Exact Sciences report-based binary criteria, or composite scores incorporating Ki-67-may yield different discordance estimates, and our findings should not be extrapolated uncritically to settings that use other clinical risk definitions.

A secondary analysis of TAILORx [5] suggested an absolute chemotherapy benefit in premenopausal women aged 50 years or younger with an RS of 16-25, and treatment decisions in this subgroup therefore continue to require individualization. In our cohort, several patients within this subgroup received chemotherapy, suggesting that age and menopausal status were carefully integrated into the decision-making process. Further subanalyses stratified by menopausal status and Ki-67 will be required in larger cohorts.

Beyond the binary decision of whether to administer adjuvant chemotherapy, the RS may also inform the choice of chemotherapy regimen in patients for whom cytotoxic therapy is indicated. A recent large real-world analysis by Chen et al. demonstrated that, among genomic high-risk (RS ≥ 31), node-negative, HR-positive/HER2-negative breast cancer patients, receipt of an anthracycline-and-taxane sequential regimen (T-AC) was associated with a significantly higher five-year distant recurrence-free interval than docetaxel plus cyclophosphamide (T-AC: 96.1% vs. TC: 91.0%), whereas no such benefit was observed at RS < 31 [15]. This finding is concordant with earlier post-hoc analyses of NSABP B-28 suggesting that the magnitude of anthracycline and taxane benefit increases with rising RS [21]. In our cohort, all patients who received adjuvant chemotherapy were treated with an anthracycline-and-taxane sequential regimen-ddEC → ddPTX in those aged ≤ 65 years, and conventional every-three-week EC followed by triweekly docetaxel (75 mg/m² × 4 cycles) in older patients-rather than with TC or an RS-stratified regimen. Although our sample size does not permit evaluation of the differential effect of regimen choice by RS category, this emerging evidence supports the future prospective integration of RS-guided regimen selection into our institutional protocol, and represents an important direction for real-world Japanese practice.

Five patients (4.9%) in our cohort were classified as clinical low/RS high - a group that would have been underestimated by clinical criteria alone but was upgraded by genomic testing. Although only one of these patients ultimately received chemotherapy, the clinical course and outcomes of this subgroup warrant careful follow-up.

Our study has several limitations. First, it was a single-center, retrospective study, and selection bias cannot be excluded, particularly with respect to the institutional indications for ordering the assay and the exclusion of patients with poorly documented histological grade. Second, the sample size (n = 102) was modest, particularly within certain subgroups (e.g., only 12 patients in the clinical high/RS high stratum and five in the clinical low/RS high stratum), which limits the statistical precision of the subgroup estimates; wider confidence intervals should be interpreted accordingly. Third, the analysis was descriptive, and no formal between-group hypothesis testing or multivariable adjustment (e.g., logistic regression for factors independently associated with the chemotherapy decision) was performed. Consequently, the independent contribution of the RS, patient preference, physician experience, and organizational factors to the treatment decision cannot be disentangled. Fourth, the follow-up period was too short to permit analysis of invasive disease-free survival or overall survival, and it therefore remains unknown whether the observed reduction in chemotherapy use translates into equivalent long-term oncologic outcomes. Fifth, the final chemotherapy decision is influenced by non-genomic factors-including patient preference, comorbidities, and social background-so the independent contribution of the RS to decision-making cannot be precisely isolated. Sixth, although we used the Adjuvant! Online version 8.0 simplified criteria adopted in MINDACT for clinical risk classification, individual Adjuvant! Online numerical scores were not calculated, and the resulting discordance rate is therefore framework-dependent. Seventh, pathology was not centrally reviewed, although biomarker assessment followed current ASCO/CAP and International Ki-67 Working Group recommendations. Eighth, the findings originate from a single Japanese academic institution and have not yet been externally validated; generalizability to other Japanese centers and to non-Japanese populations therefore requires prospective multicenter confirmation. Finally, missing data were handled exclusively by exclusion at the selection stage; given the small number of excluded patients, major bias appears unlikely but cannot be entirely ruled out. Notwithstanding these limitations, our study provides quantitative real-world evidence on the impact of the Oncotype DX assay in a histologically and pathologically uniform Japanese IDC cohort following national reimbursement.

## Conclusions

In this real-world, single-institution analysis of Japanese patients with IDC, adjuvant chemotherapy was omitted in 77.3% (95% CI, 65.7-85.7) of clinically high-risk patients following Oncotype DX testing. Oncotype DX testing was associated with a substantial change in adjuvant treatment decisions in routine Japanese clinical practice. Because the present analysis addressed decision impact rather than oncologic outcomes, the long-term safety of the observed de-escalation cannot be inferred from these data. Continued case accumulation with longer follow-up and multicenter validation are warranted to confirm the durability and safety of these decisions across broader Japanese practice.
